# A situation analysis of inter-professional education and practice for ethics and professionalism training at Makerere University College of Health Sciences

**DOI:** 10.1186/s13104-015-1577-y

**Published:** 2015-10-23

**Authors:** Pauline Byakika-Kibwika, Annet Kutesa, Rhona Baingana, Christine Muhumuza, Freddy Eric Kitutu, Catherine Mwesigwa, Rose Nabirye Chalo, Nelson K. Sewankambo

**Affiliations:** Department of Medicine, School of Medicine, Makerere University College of Health Sciences, P.O. Box 7072, Kampala, Uganda; School of Health Sciences, Makerere University College of Health Sciences, Kampala, Uganda; School of Biomedical Sciences, Makerere University College of Health Sciences, Kampala, Uganda; School of Public Health, Makerere University College of Health Sciences, Kampala, Uganda

**Keywords:** Inter-professional education, Practice, Ethics, Professionalism, Makerere University

## Abstract

**Background:**

Students at Makerere University College of Health Sciences (MakCHS) are introduced to ethics and professionalism using the inter-professional education (IPE) model. Ethics and professionalism should be running themes throughout succeeding years of study during which students are expected to develop qualities and skills for future inter-professional practice (IPP). We performed a situation analysis of IPE and IPP among students and teaching health professionals at MakCHS to guide development of a relevant training curriculum of ethics and professionalism.

**Methods:**

A cross sectional study with quantitative and qualitative methods which included questionnaires, focus group discussions and key informant interviews.

**Results:**

We interviewed 236 undergraduate students (148, 63 % male) and 32 teaching health professionals (25, 78 % male). Two hundred fifteen (91 %) students indicated they had joint learning activities with students of other professions and 166 (70 %) stated there was benefit in having an IPE model training curriculum. Most students (140, 59 %) strongly agreed that learning with other students will make them more effective members of the health team. Whereas the respondents reported inter professionalism as being well articulated in their course curricula, more than half said IPE is only implemented in the pre-clinical years of study. They noted that IPE and IPP concepts were not well programmed, health professionals engaged in teaching had poor attitudes towards IPE and IPP, there were limited numbers of skilled health care workers to implement IPP and there was poor communication between students and teaching health professionals. Majority of teaching health professionals noted challenges in implementation of IPE such as poor coordination and large student population and major factors influencing ethics and professionalism in healthcare such as limited government support, low pay for the health care workers, disrespect and lack of appreciation of the health workers by the public.

**Conclusions:**

Our findings demonstrate that IPE, IPP, ethics and professionalism are not emphasized in the clinical years of study at MakCHS. We recommend increased sensitization on the concepts of IPE and IPP plus enhanced mentorship for both students and teaching health professionals. Innovative strategies of implementation of IPE and IPP for training in ethics and professionalism must be introduced.

## Background

There is recognition that it is no longer enough for health workers to be professional but that they also need to be interprofessional [1]. One of the ten recommendations of the Commission on education of health professionals for the 21st Century is the “promotion of interprofessional and transprofessional education that breaks down professional silos while enhancing collaborative and non-hierachical relationships in effective teams” [2]. Evidence has accumulated to the effect that interprofessional education (IPE) enables effective collaborative practice which in turn enhances the quality of health-services delivery, strengthens health systems and improves health outcomes [3–6]. The IPE model brings together students from two or more professions in health during all or part of their training to learn about, from and with each other which leads to creation of a shared understanding and synergy. The aim of IPE is to equip learners with the knowledge and skills they need to work effectively as part of a health care team providing client- or patient-centered health care. Increasingly many agencies and stakeholders recognize IPE as one of the innovative strategies that will play an important role in addressing the global workforce crisis [1, 2].

In its report the Inter-professional Education Collaborative Expert panel on core competencies for inter-professional collaborative practice defined a set of four domains of required competences. The report which was inspired by the vision of IPP as the key to safe, high quality, accessible, patient care desired by all [7], states that “achieving that vision for the future requires continuous development of inter-professional competencies by health professional students through interactive learning, so that they enter the workforce ready to practice effective teamwork and team based care.”

There is adequate evidence that the hidden curriculum confers a powerful influence on the values and attitudes of students as they observe the norms, culture, behaviors and interactions in the training environment provided by the health care delivery system. Trainees’ acquisition of negative attitudes towards other health professionals has been shown to result in part from the influence of attitudes expressed by their tutors and clinicians [8]. The healthcare professional is expected to be committed to a set of values and principles that are altruistic and to put these at the heart of their practice. Acquisition of knowledge, skills and appropriate attitudes of ethics and professionalism begins before or at entry to a health professional training institution, should be emphasized during training and continue into practice. The training institutions need to be sensitive to the needs of communities in order to equip trainees with the necessary biomedical knowledge, healthcare practice, social and humanistic skills, as well as the appropriate attitudes, ethical and professional behavior [9]. Trainers need to be ethical and professional and the training curricula should emphasize the principles of ethics and professionalism.

In Uganda there is increasing concern regarding the standards of ethics and professionalism of health care providers. Despite the lack of publications on ethics and professionalism in healthcare in the country, there are increasing reports in the media of unethical and unprofessional practice. Alleged cases of absenteeism from work, negligence and extortion of money from patients in critical condition have been reported in public health facilities. This conduct substantially affects the health care system with effects such as increased morbidity and mortality and lack of trust from the public. There has been intensified effort to address the growing problem of unethical and unprofessional practice in healthcare with increased monitoring and sensitization by the Uganda Medical and Dental Practitioners’ Council and other health professional councils.

In 2013, Makerere University College of Health Sciences (MakCHS), transformed the curricula of five of its undergraduate programs, namely Bachelor of Medicine and Surgery (MBChB), Bachelor of Dental Surgery (BDS), Bachelor of Science in Nursing (BScN), Bachelor of Pharmacy (BPH) and Bachelor of Medical Radiography (BMR) from the traditional lecture-based model to a competency-based, student-centred, problem based and integrated model with inter-professional education (IPE) and community-based education and service, early clinical exposure and electives, as an approach to improve training as well as strengthen ethics and professionalism. A needs assessment of the MBChB and BScN curricula demonstrated that while it specified the competencies students were supposed to acquire during training, these competencies were not adequately integrated into the teaching and learning of individual courses [10]. In 2011 professionalism and ethical practice were identified as part of the minimum competencies for health professional education and were re-integrated into the curricula of these programs [11]. We performed a situation analysis regarding the status of IPE and IPP as perceived by students and teaching health professionals at MakCHS to guide the development of a relevant IPE training curriculum of ethics and professionalism for IPP at MakCHS.

## Methodology

### Study setting

The study was conducted at MakCHS and Mulago National Referral and Teaching Hospital. MakCHS is the largest health professional training institution in Uganda with training programs in a variety of health care disciplines (11 undergraduate and 15 postgraduate) for about 2000 students annually. The College operates in partnership with Mulago National Referral and Teaching Hospital which has nearly 1500 bed capacity. IPE is implemented especially in the first 2 years of study.

### Study design

This was a cross sectional study with both quantitative and qualitative data collection methods which included pre-tested self-administered questionnaires, focus group discussions (FGDs) and key informant interviews (KIIs).

### Study population and procedures

We enrolled a convenient sample of 250 undergraduate students and 50 teaching health professionals from the various disciplines who received a copy of the self-administered questionnaire to complete. Some of the teaching health professionals were interviewed as KIIs if they agreed and were available. The undergraduate students were selected from each year of study by systematic sampling using student lists stratified by gender and program of study. Each student completed a copy of the self-administered questionnaire. We used the Interdisciplinary Education Perception Scale [12] to test for the following subscales; competency and autonomy, perceived need for cooperation and perception of actual cooperation. Participants’ attitude to IPE was assessed basing on the following subscales; teamwork and collaboration, professional identity, roles and responsibilities [13]. Questionnaires were distributed and collected by the research assistants (RAs). We constituted two focus groups of 10–12 students each, one group with the preclinical and another with the clinical year students. We purposively identified and selected faculty for the KIIs and students for the Focus Group Discussion (FGDs) who were able and willing to discuss the subject of study. All these discussions and interviews were conducted in English language, audio recorded and transcribed verbatim without losing meaning. To ensure data quality, the question guide was pre tested and improved before data collection. Two experienced and trained research assistants (RAs) without supervisory relationship to the students or faculty facilitated the FGDs and KIIs. One RA facilitated the discussion/interview using a discussion/interview guide highlighting key issues and the second took notes. The discussion/interview guides addressed the following key issues: respondents’ understanding of IPE and IPP, ethics and professionalism and factors thought to influence implementation of IPE, IPP, ethics and professionalism. Some of the questions asked during the FGD were; (1) What do you understand by the term IPE? (2) In your view, is the IPE approach applied during undergraduate training at MakCHS? (3) How would you describe the practice of IPE at MakCHS? (4)What is your opinion about IPE during the training of undergraduates? (5) What changes would you recommend to improve the current approach to IPE at MakCHS? (6) What do you understand by the term IPP? (7) Is IPP being implemented in the teaching health centers? (8) What factors affect IPP in health facilities? (9) What are the benefits of IPP?

Data from FGDs and KIIs were analyzed separately and manually where by the typed transcripts were read several times to obtain key emerging issues on which basis data were categorized. Initial categories were drawn from the interview guide and transcripts while we allowed for open coding to obtain further patterns as they emerged after thoroughly reviewing the data. The categories which emerged were grouped into the following key themes; knowledge, perception and status of IPE and IPP, ethics and professionalism and factors thought to influence implementation of IPE, IPP, ethics and professionalism. Quantitative data analysis was performed using SPSS version 14.0 and included data summary into frequencies, medians and interquartile range.

### Ethical considerations

The study protocol was reviewed and approved by Makerere University School of Health Sciences Research and Ethics Committee and Uganda National Council for Science and Technology. The RAs explained the study to participants, assured them of confidentiality and anonymity and obtained written informed consent. Participants were assigned study numbers. Names and other identifying information were not included on the data collection instruments. Data collection tools were stored separately from the signed consent forms in locked cupboards. Participants were given verbal assurance that this information would be used for study purposes only without revealing their identity. To ensure confidentiality the audio records were kept in locked cupboards only accessible to the study team.

## Results

### Description of student study participants

A total of 236 undergraduate students, of whom 148 (63 %) were male, completed the self-administered questionnaires. Description of students’ study program and year of study are shown in Table 1. Two hundred fifteen (91 %) students said they had joint learning activities with students of other professions and 166 (70 %) said there was benefit in having a pre-service training curriculum of the IPE model.

**Table 1:** Description of students’ study program and year of study

Study program (N = 236) n (%)		Year of study (N = 236) n (%)	
MBChB	90 (38)	First	64 (27)
BPH	40 (18)	Second	66 (28)
BScN	39 (16)	Third	73 (31)
BDS	37 (15)	Fourth	22 (9)
BMR	30 (13)	Fifth	11 (5)

### Students’ knowledge, attitudes and perceptions of IPE and IPP

Most students 140 (59.3 %) strongly agreed that learning with other students will make them more effective members of the health team. Table 2 is a summary of students’ readiness and attitudes towards IPE. Two FGDs were conducted, one with pre-clinical students and another with clinical students, each lasting 90 to 120 min. Nearly all participants of the FGDs were knowledgeable of the concepts of IPE and IPP. Majority of them indicated that they had heard about the IPE concept and could easily define the term IPE. They reported that IPE is well articulated in year one of their studies. The main elements of IPE and IPP identified included collaborative learning and training that cuts across different professions, as quoted below.

**Table 2:** Students’ readiness and attitudes towards IPE

	Strongly disagree (1)	Disagree (2)	Neutral (3)	Agree (4)	Strongly agree (5)
Learning with other students will help me become a more effective member of a health care team	17 (7.2)	7 (3.0)	7 (3.0)	65 (27.5)	140 (59.3)
Patients would ultimately benefit if health care students worked together to solve patient problems	19 (8.1)	6 (2.5)	8 (3.4)	58 (24.6)	145 (61.4)
Shared learning with other health care students will increase my ability to understand clinical problems	16 (6.8)	10 (4.2)	15 (6.4)	75 (31.8)	120 (50.8)
Learning with other health care students before qualification would improve relationships after qualification	17 (7.2)	10 (4.2)	10 (4.2)	77 (32.6)	122 (51.7)
Communication skills should be learned with other health care students	17 (7.2)	9 (3.8)	21 (8.9)	84 (35.6)	105 (44.5)
Shared learning will help me to think positively about other professionals	17 (7.2)	12 (5.1)	14 (5.9)	93 (39.4)	100 (42.4)
For small group learning to work, students need to trust and respect each other	19 (8.1)	8 (3.4)	13 (5.5)	80 (33.9)	116 (49.2)
Team-working skills are essential for all health care students to learn	20 (8.5)	6 (2.5)	8 (3.4)	75 (31.8)	127 (53.8)
It is not necessary for undergraduate health care students to learn together	114 (48.3)	68 (28.8)	21 (8.9)	7 (3.0)	26 (11.0)
Shared learning will help me to communicate better with patients and other professionals	12 (5.1)	13 (5.5)	28 (11.9)	104 (44.1)	79 (33.5)
Shared learning will help to clarify the nature of patient problems	9 (3.8)	15 (6.4)	37 (15.7)	107 (45.3)	68 (28.8)
Shared learning before qualification will help me become a better team worker	17 (7.2)	4 (1.7)	18 (7.6)	81 (34.3)	116 (49.2)

*“I understand IPE as the system where various professionals are training as a team so working as a team, they keep supplementing each other, they keep teaching each other the skills that probably the other profession is not conversant with in order to have an overall goal achieved and these may include student doctors, student nurse, student radiologist, student pharmacist and all these people come together to supplement each other and teach each other.” FGD pre*-*Clinical students*.

*‘‘IPE is learning and doing activities jointly and collaboratively’’ BScN student, yearII.*

Almost all respondents in the FGDs stated that it is beneficial to have an IPE based pre-service training curriculum. The main benefits mentioned included; imparting confidence in the different professions, gaining a wider perspective in inter professional training, more knowledge and skills, improving specialization and exposure to conditions under which the students will practice after graduation and simplifying work, as cited below.

*“IPE gives us actual exposure to the real conditions in which we have to serve in as health care providers and we get a wider overview of what we are supposed to do” BScN student, year I.*

Similar to IPE, majority of the respondents reported having heard of IPP and could identify some of the benefits of IPP during health care delivery such as; improving relationships, communication skills and better service delivery, building confidence in different areas of specialty, better understanding and efficiency in patient management, encouraging holistic management of patients, team work and respect for one another, as cited below.

*“IPP encourages team work which later produces positive results that will improve health care” MBChB student, year III.*

*“IPP provides more holistic health care services’’ MBChB student, year II.*

*“IPP gives us wider knowledge hence improved patient care’’ BPH student, year I.*

*“….holistic care, the work we handle becomes less burdensome and there are better health outcomes… ’’FGD Clinical students.*

Additional benefits of IPP and IPE mentioned included: effective service delivery, better health outcomes, holistic care to patients, making work easy, saving time, and addressing problems of under staffing, improving relationships between students and teaching health professionals, team work, promotion of ethical practice between and within the different professions and better health outcomes.

### Students’ perception of the current status of IPE and IPP

Variations were noted in the way students perceived the level of implementation of IPE and IPP in their training. Whereas the respondents reported inter professionalism as being well articulated in their course curricula, more than half said IPE is only implemented in the pre- clinical years of study, while majority perceived IPE and IPP implementation as having some gaps. They noted that IPE and IPP concepts were not well programmed, there was limited number of skilled health care workers to implement IPP, health workers lacked motivation and there was poor communication between teaching health professionals and students. There were mixed views as to whether IPP was being practiced.

Students identified the main gaps in IPE and IPP implementation as large numbers of students with limited space at the college, time constraints, administrative and course assessment gaps, competing course demands, limited funding, lack of integrated learning materials, a feeling of superiority and inferiority of some students taking particular courses, assessment challenges and lastly supervision challenges. Some respondents thought that IPE was not properly designed therefore giving the impression that they were forced to attend course units that were irrelevant to their primary programs of study, as cited below.

“*For us the dental surgery students, we have not started learning anything about dentistry, so we all learn irrelevant and unnecessary things like the anatomy of the leg and embryology as a course unit*” BDS student.

*“Some of the activities seem out of your profession, at the moment for example BDS students are rotating in pediatric and maternity wards” BDS student.*

### Students’ perceptions on factors influencing ethics and professionalism in healthcare

Students highlighted factors influencing ethics and professionalism in healthcare as teaching health professionals’ poor attitudes, limited government support, patients’ ignorance of their rights and where to report when they are violated, low pay for the health care workers, disrespect and lack of appreciation of the health workers by the public, as quoted below.

*“Unprofessionalism is a big problem, considering that health personnel are not paid their worth so they resort to other means of surviva’” MBChB student, year III.*

*“Unprofessionalism is due to limited resources even within departments, poor culture and hostility of the general public and the rampant poverty that is prevailing lures professionals into corrupt business to earn a living” BPH student, year I.*

### Description of teaching health professional participants

Thirty-two teaching health professionals from both pre-clinical and clinical departments completed the self-administered questionnaires. Twenty-five (78 %) were male.

The median, inter quartile range (IQR) of age and duration in teaching service were 39 (32.7–43.2) and 8 [3–10] years. Seven (22 %) had doctorate degrees, 23 (72 %) masters degree, 1 (3 %) had completed a fellowship and 1 (3 %) had a bachelor’s degree. Twelve (36 %) were lecturers, 1 (3 %) professor, 2 (6 %) associate professors, 4 (12 %) senior lecturers, 7 (22 %) assistant lecturers and 6 (19 %) teaching assistants. The KIIs lasted 30 to 60 min. Only 11 (34 %) said they had heard of IPE and IPP before and could define and list benefits of IPE and IPP. Teaching health professionals’ ranking of emphasis placed on the different competencies during training is shown in Table 3.

**Table 3:** Teaching health professionals’ ranking of emphasis put on the different competencies during training

Competence	Ranking				
	Not at all emphasized n (%)	Slightly emphasized n (%)	Mentioned n (%)	Emphasized n (%)	Do not know n (%)
Team work	0	2 (6)	7 (22)	21 (66)	2 (6)
Respect for other health professions	0	5 (16)	7 (22)	18 (56)	2 (6)
Learning the roles of other health professions	0	5 (16)	8 (25)	16 (50)	3 (9)
Ethics	1 (3)	2 (6)	8 (25)	19 (60)	2 (6)
Appreciation of your profession	0	2 (6)	5 (16)	4 (18)	9 (3)
Acquisition of practical skills	0	2 (6)	3 (5)	18 (56)	3 (9)

### Teaching health professionals’ perception of the status of IPE and IPP at MakCHS

Majority of the KIIs who knew about IPE observed that IPE was well established although with challenges such as poor coordination, large number of students, limited health professionals and lack of faculty commitment to the IPE concept among others. Some of their responses are cited below;

*“IPE refers to participation in several learning activities as a group of professionals or students who will in practice work side by side, as a team or in the same environment.” KII from the Department of Family Medicine.*

“…*at the moment it is well established although it has its own challenges in terms of student numbers, the content of information being given to the students, being able to balance the content to suit the different professionals is a challenge to the lecturers as well as the students, the classes are usually big and it becomes a problem keeping them together to see that they have actually learnt something just because of the numbers” KII from the Department of Surgery.*

Harmonizing the needs of the different professionals during IPE was cited as a big challenge coupled with the lack of integrated learning materials to support IPE. Other challenges mentioned by teaching health professionals included lack of training in IPE, difficulty in making time tables, supervision, assessment, facilitation and infrastructural challenges, as cited below.

*“Each group has different needs and learning objectives. So it is hard to harmonize and conduct a singular group session” KII from the Department of Psychiatry.*

*“Timing is a problem because the curricula are conducted at different times for different disciplines hence mismatches” KII from the Department of Speech and Language Therapy.*

*“Assessment should also be integrated to demonstrate the relevance of the various disciplines in patient care” KII from the Department of Pharmacy.*

Respondents from all departments reported that IPE improves team work and contributes to appreciation of other professions as cited below.

*“IPE improves teamwork in provision of health care. Team members appreciate each other’s roles on healthcare team” KII from the Department of Pharmacy.*

### Teaching health professionals’ perceptions on factors affecting ethics and professionalism

Teaching health professionals mentioned factors such as lack of knowledge, people’s negative attitudes, limited human resource and inferiority complex among some health professionals as major factors affecting IPE, IPP, ethics and professionalism.

*“I think there is lack of respect for each others’ professions where if all would consult each other it would benefit the patient. The college has done its part but the practitioners’ mind set is totally different, their first priority is survival this is a very dangerous phenomena, in the end it hurts the patient” KII from the Department of Obstetrics and Gynecology.*

*“What we train as the ideal in IPE is not what the learners find out there in practice because of inequalities or poor distribution of professionals. The distribution is controlled by other factors which are beyond our control” KII from the Department of Family medicine.*

The gaps in the current training methods of ethics and professionalism were largely attributed to limited resources across all the departments including limited number of role models and mentors, shortage of learning materials such as textbooks, limited practical activities and inadequate supervision. Teaching health professionals said ethics was mainly taught in the pre-clinical years of study as cited below;

*“Ethics is taught at the beginning of the program and for some programs there is less teaching towards the end. It should be continuous” KII from the Department of Pharmacy.*

## Discussion

We performed a situation analysis of IPE and IPP among students and teaching health professionals at MakCHS to be able to guide the development of a relevant IPE-based curriculum for ethics and professionalism education. Our study shows that students and teaching health professionals are knowledgeable about the concepts and benefits of IPE and IPP. However, the major challenge lies in the implementation of IPE and IPP. Whereas the concepts are well articulated in the training curriculum, implementation is unsatisfactory. There is better implementation in the pre-clinical but less so in the clinical years of study yet students are more likely to adopt experiences gained during clinical years of study for application during their post-training practice.

We used previously developed and validated tools for data collection which included; the Interdisciplinary Education Perception Scale, developed and validated by Luecht et al., and modified by Mcfadyen et al., in 2007 [12] to test for the following subscales; competency and autonomy, perceived need for cooperation and perception of actual cooperation. Participants’ attitude to IPE was assessed by a tool developed by Glennys Parsell and John Bligh in 1999 [13]. FGDs and KIIs were deemed the most appropriate qualitative methods for data collection in this study owing to the non-sensitivity of the subject and experiences of faculty, clinical and preclinical year students required. We think that these methods yielded the best possible responses from out participants, despite the limitation of having one FGD each with preclinical and clinical year students. Members of FGDs can vary both in experience and opinion, however, members of our two FGDs were in agreement and the reports yielded similar results.

Major factors highlighted as hindering IPE and IPP were also reported to influence the practice of ethics and professionalism in the country. This article contributes insights into many of the challenges faced in the design and implementation of the IPE and IPP approach to strengthening ethics and professionalism at the largest health training institution in the country. The identified factors are multifactorial and will require a multi-pronged approach with emphasis on aspects such as improving the work environment and remuneration, sensitization of health consumers, increased awareness of health rights and access to medico-legal services, good leadership and commitment, coordination, increasing availability of role models, and resources including trained trainers who are ethical, professional and competent in IPE and IPP models. Our data highlight the critical need and importance of training teaching health professionals on the concepts of IPE and IPP for collaborative practice.

Over the years, there have been lots of changes in the training of health professionals as well as changes and challenges in their work settings. In addition there has been increase in the prevalence of chronic diseases such as HIV and non-communicable diseases, longer treatment duration and disease and drug related complications and interactions which require IPP for effective management. Our findings are in agreement with previous reports that make the case for strengthened IPE and IPP as a tool for achieving the “triple aim” of better patient care, better health outcomes and more efficient and affordable education and health care systems [14] as solutions to healthcare challenges in Uganda and Africa [15]. Recommendations from expert panels include ethics for IPP as a core competence for collaborative practice and several universities are currently implementing the student-centered, problem based and integrated learning model and IPE to facilitate health professions students’ acquisition of these competencies [7]. The IPE approach allows students from two or more professions to learn about, from and with each other and prepares them for future IPP [14]. In Uganda, some trainers including a consortium of Uganda medical schools and the Medical Education Partnership for Equitable Services to all Ugandans (MEPI) identified ethics and professionalism among others as key gaps in ideal graduate competencies required to address the health needs in the country [9, 16] and have already initiated some innovative strategies to address these gaps. It is hoped that strengthening these innovative strategies will enhance development of competencies for ethics and professionalism [9, 17].

Our analysis highlights several challenges in implementing IPE and IPP, however, many of them are logistical and can be solved if the leaders and health professionals are strongly committed to better health care. A critical message is the issue of role models and leaders, good planning and training of implementers, as well as commitment from all stakeholders. The findings are in agreement with a previous exploratory study at MakCHS which described the formal curriculum of teaching professionalism as being inadequate while the hidden and informal curricula plays a critical role in learning. In that study, students identified role models as being essential to their development of professionalism and emphasized the need for appropriate role modelling [18]. A needs assessment for mentorship conducted at MakCHS revealed almost similar challenges [19]. These reports have caused enhanced efforts to expand mentorship at MakCHS with establishment of a formal mentorship program with the hope for improved career, ethics and professional development and training.

## Conclusion

Our findings demonstrate that IPE, IPP, ethics and professionalism are part of the existing curricula at MakCHS, however, they are emphasized in the pre-clinical years of study and less implemented in the clinical years of study. Several logistical challenges affect implementation of IPE and IPP for training in ethics and professionalism. We recommend increased sensitization and training on the concepts of IPE and IPP plus enhanced mentorship for both students and teaching health professionals. Innovative strategies of implementation of IPE and IPP for training in ethics and professionalism must be introduced in the training curricula of health professionals at MakCHS.

## Abbreviations

IPE: inter-professional education
IPP: inter-professional practice
MakCHS: Makerere College of Health Sciences

## References

[1] World Health Organization WHO (2010). Framework for action on interprofessional education & collaborative practice.

[2] Frenk J, Chen L, Bhutta ZA, Cohen J, Crisp N, Evans T (2010). Health professionals for a new century: transforming education to strengthen health systems in an interdependent world. Lancet.

[3] Holland R, Battersby J, Harvey I, Lenaghan E, Smith J, Hay L (2005). Systematic review of multidisciplinary interventions in heart failure. Heart.

[4] Reeves S, Zwarenstein M, Goldman J, Barr H, Freeth D, Hammick M, Koppel I. Interprofessional education: effects on professional practice and health care outcomes. Cochrane Database Syst Rev. 2008;(1):CD002213. doi:10.1002/14651858.CD002213.pub2. 10.1002/14651858.CD002213.pub2 18254002

[5] Cooper H (2001). Developing an evidence base for interdisciplinary learning: a systematic review. J Adv Nurs.

[6] Barr H (2000). Evaluations of interprofessional education: a United Kingdom review for health and social care.

[7] Interprofessional Education Collaborative Expert Panel (2011). Core competencies for interprofessional collaborative practice: report of an expert panel.

[8] Leaviss J (2000). Exploring the perceived effect of an undergraduate multiprofessional education intervention. Med Educ.

[9] Kiguli S, Mubuuke R, Baingana R, Kijjambu S, Maling S, Waako P (2014). A consortium approach to competency-based undergraduate medical education in Uganda: process, opportunities and challenges. Educ Health.

[10] Kiguli S, Baingana R, Paina L, Mafigiri D, Groves S, Katende G (2011). Situational analysis of teaching and learning of medicine and nursing students at Makerere University College of Health Sciences. BMC Int Health Hum Rights..

[11] MESAU Consortium. MESAU consortium defines the minimum competencies for medical education in Uganda. Kampala 2011 [cited 2012 26 August 2012]. http://www.chs.mak.ac.ug/content/mesau-consortium-defines-minimum-competencies-medical-education-uganda.

[12] McFadyen AK, Maclaren WM, Webster VS (2007). The Interdisciplinary Education Perception Scale (IEPS): an alternative remodelled sub-scale structure and its reliability. J Interprof Care.

[13] Parsell G (1999). Bligh J. The development of a questionnaire to assess the readiness of health care students for interprofessional learning (RIPLS). Med Educ.

[14] Gilbert JH, Yan J, Hoffman SJ (2010). A WHO report: framework for action on interprofessional education and collaborative practice. J Allied Health.

[15] Manabe YC, Campbell JD, Ovuga E, Maling S, Bollinger RC, Sewankambo N (2014). Optimisation of the Medical Education Partnership Initiative to address African health-care challenges. Lancet Glob health.

[16] Kiguli S, Baingana R, Paina L, Mafigiri D, Groves S, Katende G (2011). Situational analysis of teaching and learning of medicine and nursing students at Makerere University College of Health Sciences. BMC Int Health Hum Right.

[17] Nshaho J (2005). Innovative strategies in teaching of biomedical sciences to health professionals. Niger J Physiol Sci.

[18] Baingana RK, Nakasujja N, Galukande M, Omona K, Mafigiri DK, Sewankambo NK (2010). Learning health professionalism at Makerere University: an exploratory study amongst undergraduate students. BMC Med Educ.

[19] Damalie N, Byakika-Kibwika P, Kintu K, Aizire J, Nakwagala F, Luzige S, Namisi C, Mayanja-Kizza H, Kamya MR (2011). Mentorship needs at academic institutions in resource-limited settings: a survey at makerere university college of health sciences. BMC Med Educ.

